# IMPACT OF THE CRITICAL CARE PATHWAY ON INTRINSIC CAPACITY ON HEALTHY AGING PROMOTION

**DOI:** 10.1093/geroni/igae098.0546

**Published:** 2024-12-31

**Authors:** Xiangyu Zhai, Yiting Tang, Shuangzhou Chen, Feng Chen, Doris Sau Fung Yu

**Affiliations:** The University of Hong Kong, Hong Kong, China (People’s Republic); The University of Hong Kong, Hong Kong, China (People’s Republic); The University of Hong Kong, Hong Kong, China (People’s Republic); The University of Hong Kong, Hong Kong, China (People’s Republic); The University of Hong Kong, Hong Kong, China (People’s Republic)

## Abstract

The United Nations Decade of Healthy Aging prompted the implementation of the “Pathway to Healthy Aging”, a 12-week health-social collaborative care program. This program, based on the Integrated Care for Older People (ICOPE) care pathway by the World Health Organization, incorporated clinical critical pathways, empowerment-based behavior modification, and health-oriented peer support. Intrinsic capacities of older adults were used as health indicators for effectiveness evaluation. From January 2023 to January 2024, 1024 older adults (female = 868; Mage = 75.5, SD = 7.52) participated, with 493 completing a six-month follow-up. Outcome-based evaluations demonstrated significant improvements across three time-points in locomotor capacity based on Timed Up and Go Test (χ²(6)=73.49, p<0.001, Cramér’s V=0.16) and Short Physical Performance Battery (χ²(4)=31.83, p<0.001, Cramér’s V=0.12), cognitive capacity (measured by Montreal Cognitive Assessment 5-minute protocol; F(2, 984)=52.93, p<0.001, partial η²=0.11), and psychological capacity (measured by 15-item Geriatric Depression Scale; F(2, 984)=11.31, p<0.001, partial η²=0.09). No significant change in hand grip strength (χ²(4)=6.15, p=0.188). Vitality capacity, based on Mini Nutritional Assessment, improved immediately after the program (χ²(4)=7.45, p=0.047, Cramér’s V=0.09) but showed no significant further change at the six-month follow-up, based on pairwise comparison analyses (Bonferroni’s correction). Insomnia (The Insomnia Severity Index) also significantly improved (χ²(6)=77.42, p<0.001; Cramér’s V=0.16). This program demonstrates the effectiveness of the ICOPE care model in promoting healthy aging in real-world settings, emphasizing the potential of collaborative care models to enhance the elderly population’s intrinsic capacities. These findings have critical implications for stakeholders working towards the United Nations’ goal of fostering healthy aging.

